# The Psychological Effect of Internet-Based Mindfulness-Based Stress Reduction on the Survivors of Breast Cancer During the COVID-19

**DOI:** 10.3389/fpsyt.2021.738579

**Published:** 2021-09-30

**Authors:** Chuanyuan Kang, Shufang Sun, Zhuangqing Yang, Xinxin Fan, Jing Yuan, Li Xu, Yujun Wei, Huiqi Tong, Jianzhong Yang

**Affiliations:** ^1^Department of Psychosomatic Medicine, Tongji University School of Medicine, Shanghai East Hospital, Shanghai, China; ^2^Mindfulness Center at Brown University, Department of Behavioral and Social Sciences, Brown University School of Public Health, Providence, RI, United States; ^3^The 3rd Department of Breast Surgery, Breast Cancer Centre of Yunnan Cancer Hospital, The Third Affiliated Hospital of Kunming Medical University, Kunming, China; ^4^Department of Psychiatry, The Second Affiliated Hospital of Kunming Medical University, Kunming, China; ^5^Department of Psychiatry and Behavioral Sciences, Stanford University, Stanford, CA, United States

**Keywords:** breast cancer survivors, internet-based mindfulness-based stress reduction, efficacy, engaged time, COVID-19

## Abstract

**Objective:** To examine the efficacy and the role of engagement of an internet-based Mindfulness-based Stress Reduction (iMBSR) for survivors of breast cancer (BC) during the COVID-19 period from January to March in 2020 in China.

**Methods:** 48 survivors of BC were divided into the absentees group and the iMBSR groups according to their attending to the standardized, group-based, 8-week iMBSR. Based on practice time, survivors of BC in the iMBSR were categorized into three subgroups: group 1 (<30 min/day), group 2 (30–60 min/day), and group 3 (>60 min/day). In addition, participants were classified as partial attendees (<4 sessions) and completers (more than 4 sessions) of the iMBSR groups. All participants were evaluated for symptoms of depression, anxiety and insomnia at baseline, mid-intervention, and post-intervention.

**Results:** After an 8-week iMBSR practice, at mid-intervention and post-intervention, participants in iMBSR group had significant improvement in scores and reduction rates of depression, anxiety, and insomnia compared to absentees. Scores of depression and insomnia, reduction rates of depression at post-intervention, scores of anxiety, reduction rates of anxiety and insomnia at mid-intervention and post-intervention, had significant differences among subgroups of practice time. Daily practice time was positively related to reduction rates of depression, anxiety and insomnia at post-intervention in the iMBSR group.

**Conclusion:** Internet-based MBSR showed efficacy in reducing psychological symptoms among survivors of BC. For survivors of BC, iMBSR practice has a potential dose–response efficacy, with a threshold of >30 min daily practice for most optimal symptoms reduction.

**Trial Registration:** Registration number is [ChiCTR2100044309].

## Introduction

A report on the global burden of cancer worldwide for estimates of cancer incidence and mortality in 2018 showed that breast cancer (BC) was the second commonly diagnosed cancer, accounting for 11.6% of total cancer cases. Among females, breast cancer is the most commonly diagnosed cancer and the leading cause of cancer death (1). In China, the estimate of new breast cancer cases was about 278,900 in 2014, accounting for 16.51% of all new cancer cases in female and was also one of the most common malignant tumors threatening to women's health (2). BC survivors face challenges to cope over time with high physiological and psychological symptoms burden and distress, which affect their well-being and quality of life (3). Recently two systematic reviews and meta-analyses showed high global prevalence of depression and anxiety among BC patients (32.2 and 41.9%, respectively) (4, 5). Untreated symptoms of depression and anxiety in BC patients could lead to poor quality of life, increased mortality (6), and high economic costs (7).

Derived from Buddhist tradition, mindfulness is described as a “way of being” and defined as the capacity for awareness in each moment, by “paying attention in a particular way: on purpose, in the present moment, and non-judgmentally” (8). With an emphasis on self-regulation of attention, mindfulness can be characterized by non-judgmental moment-to-moment awareness, patience and calmness, openness and trust, non-striving, letting go, and compassion (9). Recent findings of a meta-analysis support the short-term effectiveness and safety of two prominent mindfulness-based interventions (MBIs), namely mindfulness-based stress reduction (MBSR) and mindfulness-based cognitive therapy (MBCT), for women diagnosed with breast cancer as adjuvant treatment, such that they improved patients' well-being and health related quality of life (HQoL), and reduced symptoms of fatigue, insomnia, anxiety, depression, and stress (10–13). Recently an Internet-delivered Mindfulness-Based Cognitive Therapy (iMBCT) intervention was proved to be efficacious in reducing symptoms of anxiety and depression for BC or prostate cancer survivors (14), suggesting that the internet-based MBIs could be administered to cancer survivors. However, supported evidence primarily comes from Western countries and there has been a lack of research on the utility and efficacy of MBIs in the global context for cancer survivors, such as in China. This lack of evidence hampers our understanding on the potential utility of MBIs in reducing the global mental health burden among cancer survivors.

With the outbreak of the 2019 coronavirus disease (COVID-19) (15) and its rapid widespread around the world, elevating panic, fear and psychological symptoms among the public became a common phenomenon (16, 17). Public health measures to contain and mitigate the spread of COVID-19 have been implemented worldwide, such as massive lockdown and quarantine. China, in particular, placed strict nation-wide quarantine measures (e.g., “shelter at home”) following the outbreak of COVID-19 in Wuhan, Hubei in January, 2020. However, one of the unintended consequences of quarantine is the elevated psychological symptoms among people with chronic illness (18), such as cardiovascular diseases, active cancer, diabetes, stroke, and dementia. Sudden and unexpected separation from loved ones, shortage of living supplies, the loss of freedom of moving around, and uncertainty over disease status all contribute to increased psychological distress (19). In addition, some patients have been confronted with difficulties in routine medical treatments due to delayed transportation and shortages of medicines and medical staffs in hospitals (20).

Women with BC are already at a higher risk for psychological distress, the additional stress of the pandemic may contribute to further increase their vulnerability. During the COVID-19 pandemic, psychological assistance hot-lines, online self-help intervention courses were widely utilized in China (21). Even though there are lots of online psychological self-help services, many questions remain unanswered with regards to internet-based mental health services, particularly in low and middle-income countries where demand for mental health services is high yet funding and resources lag behind (22). Adherence has been shown to be a measure for treatment's acceptability and a determinant for treatment's effectiveness (23). Unfortunately, poor adherence to depression treatment, both medication and psychotherapy, frequently interferes with treatment effectiveness (24). Although internet-based services present great opportunity in reach and scalability, efficacy and adherence of such programs during the pandemic period are largely unknown. Mindfulness was found to be a protective factor of psychological distress during the pandemic among the general public (17). However, to our best knowledge, there has not been any empirical research that evaluated the efficacy and engagement of internet-based psychological interventions for the BC patients during COVID-19 pandemic. As patients with chronic illnesses including those with BC often face multiple stressors during the pandemic, evaluating treatment efficacy and engagement to internet-based psychological interventions is key to inform mental health and integrated care for BC patients during a public health emergency.

As one of internet-delivered Mindfulness-Based Interventions (iMBIs), internet-based MBSR (iMBSR) has also shown to be efficacious in treating psychological distress among cancer patients (25). Therefore, in the current study, iMBSR was conducted among survivors of BC during the 2 months from February to March, 2021, during which the COVID-19 outbreak was announced and followed by nation-wide lockdown in February, 2021. There are two aims of the study. First, we examined the efficacy of iMBSR for survivors of BC during the COVID-19 pandemic. Specifically, we hypothesized that iMBSR would be efficacious in reducing psychological distress and improving well-being among survivors of BC during this time. Second, we explored the dose-response relationship regarding engagement with iMBSR (i.e., attendance, practice time) and efficacy. Specifically, we anticipated that higher engagement would result in better treatment outcomes.

## Materials and Methods

### Subjects

This was a 2-month single arm trial, and the registration number is [ChiCTR2100044309]. Convenience sampling was used in the present study. Women diagnosed with BC who received radical mastectomy, or modified radical mastectomy or breast-conserving surgery, and reported emotional distress were referred and recruited voluntarily to the research team following diagnosis and the completion of surgery. Inclusion criteria included: (1) female sex, (2) aged 18 years or older, (3) a diagnosis of Stage 0, I, II, or III BC, (4) treatment with a radical mastectomy, or modified radical mastectomy or breast-conserving surgery, (5) completion of adjuvant radiation and/or chemotherapy at least 2 weeks prior to enrollment and within 1 year of the completion of a primary treatment.

Exclusion criteria included: (1) evidence of cognitive impairment that prevents from meaningful participation in the study, (2) carrying a diagnosis of schizophrenia, obsessive compulsive disorder, post-traumatic stress disorder, alcohol-related diseases due to the need for specialized treatment of these psychiatric illnesses, (3) imminent risk of suicide. The last item of the 9-item Patient Health Questionnaire (PHQ-9) is on suicide ideation, if a participant rated one or above, she was then excluded from the research, (4) diagnosis of Stage IV cancer, (5) a cancer recurrence.

The study was carried out in accordance with the latest version of the Declaration of Helsinki, and was approved by the Ethics Committee of the Second Affiliated Hospital to Kunming Medical University. Informed consent of the participants was obtained after the nature of the procedures had been fully explained with opportunities to answer any questions raised by interested participants. Following informed consent, 48 interested survivors of BCattended the 8-week iMBSR program.

### Procedure

Recruitment period lasted for 3 months, from November 1th 2019 to January 31th 2020. A total of 54 survivors of BC were recruited. As planed in the research protocol, all participants completed self-administered psychological evaluation during the last week of January, 2020. However, a COVID-19 outbreak was declared officially on January 21th, 2020 by the Chinese government. The epidemic quickly began to cause a national concern and in other provinces outside Hubei, people avoided hospitals so as to prevent infection. Moreover, the strictest level of quarantine measures took effect in Wuhan on January 23th, 2020, triggering a range of quarantine policies across China including a national lockdown from January 23th through the end of March, 2020. With the sudden and fast spreading epidemic, all participants were invited to self-administer a set of questionnaires as the baseline assessment *via* a widely used online survey tool, SoJump from January 24 to 31th 2020. Chinese versions of a number of measures with established reliability and validity were used to assess symptom severity and remission status. At the end of January 31th 2020, a total of 48 participants completed the baseline assessment.

All 48 survivors of BC were invited to attend an internet-based, 8-week MBSR course, from the 8th February to 28th March *via* a widely used online video conferencing App in China (Tecent). All 48 participants were evaluated at baseline, mid-intervention (4th week), and post-intervention (8th week). Each month, 48 survivors of BC would receive the follow-up by their surgeon by telephone or online. Therefore, even though some of survivors didn't attend the course or any session, they still received the evaluation.

### Intervention Protocol

The practice of iMBSR has two components: one is an internet-based, standardized, group-based, 8-week MBSR course (9), lasting for an average of 2.5 h weekly. The other is home practice assignments, which consist of 45 min of at-home meditation practice for 6 days out of 7. All Participants were invited to attend the same class. Led by a certified MBSR instructor, weekly group sessions focused on mindfulness meditation including body scan, sitting meditation and mindful movement (Yoga) as well as small and large group discussions of participants' experiences of both in-session and home practices. A practice time record was used in order to collect data regarding participants' time allotted to mindfulness practices. Homework assignments were given throughout the course.

### Measures

Chinese versions of measures with established reliability and validity were used to assess the severity and remission status of symptoms, including depression, anxiety and sleep quality. Subjects completed three self-administered scales: the 9-item Patient Health Questionnaire (PHQ-9) (26), the 7-item Generalized Anxiety Disorder scale (GAD-7) (27), and the Pittsburgh Sleep Quality Index (PSQI) (28).

Depressive symptoms were measured through an adapted Chinese version of PHQ-9 (26). Cronbach's alpha coefficient was 0.892 with our sample, and symptom severity was defined as mild, moderate or severe using the recommended clinical cutoffs of total scores of 6, 12, and 15 respectively on the PHQ-9 (29).

Generalized anxiety symptoms were measured through an adapted Chinese version of GAD-7 (27). Cronbach's alpha coefficient was 0.93 in this sample, and symptom severity was defined as mild, moderate or severe according to recommended clinical cutoffs of total scores of 4, 9, 12, respectively on the GAD-7 (30).

Sleep quality evaluation, including insomnia, was measured through an adapted Chinese version of PSQI (28). Cronbach's alpha was 0.845 in this sample. Since there are no established severity cutoffs for the Chinese version of the PSQI, the continuous score of the instrument was used to establish severity, with higher scores indicating worse sleep quality.

In addition to the scores of PHQ-9, GAD-7, and PSQI, reduction rate was also used to evaluate the therapeutic effect of the iMBSR. Following standard practice of calculating reduction rate (31), the formula: reduction rate = (baseline score – score after intervention)/baseline score × 100% was adopted.

### Definitions

Among 48 survivors of BC enrollees, 19 of them did not attend the MBSR class, neither did they do the homework assignments, so the 19 enrollees were classified as absentees group, and the other 29 enrollees were called as iMBSR group. The reasons for absence varied from doubt in MBSR, concerns of time consumption and lack of consistent access to internet.

In the iMBSR group, the participants who attended the course <4 sessions were classified as partial attendees of iMBSR group, and those who attended at least 4 sessions out of the 8-week MBSR course were classified as completers of iMBSR group.

In this study, we characterized engagement by both attendance and home practice time. Regarding practice time, we divided iMBSR group into three subgroups of different practice times on average, including subgroup 1 (<30 min/day), subgroup 2 (30–60 min/day), and subgroup 3 (>60 min/day).

### Statistical Analysis

Statistical analyses were performed by SPSS 25.0 (Statistical Product and Service Solutions, SPSS inc). Continuous data were presented as mean ± standard deviation (SD). Categorical data were presented as absolute numbers and percentages. First, we compared demographic and clinical characteristics between the absentees group and iMBSR group with the categorical data by Fisher's exact tests and continuous data by one-way ANOVA or Kruskal-Wallis H rank sum tests. Pairwise comparisons across different evaluation months were analyzed by one-way ANOVA or Kruskal-Wallis H rank sum test. In addition, the pre-post efficacies for symptoms were analyzed by using Cohen's *d*, which has the rule of thumb of interpreting effect sizes (Cohen's *d* > 0.8 represents large effect size) (32).

Linear regression analysis was used to explore whether practice time was associated with the therapeutic effect of iMBSR. However, the analysis showed that the residuals were not normally distributed, then generalized linear models (GLMs) were utilized with the reduction rates of PHQ-9, GAD-7 and PSQI at post-intervention respectively as the dependent variable and practice group as the independent variable. We used a two-sided alpha = 0.05 for all statistical significance analysis.

## Results

### Participant Characteristics

Forty-eight survivors of BC, all women, were recruited for the study. Among them, nine participants (18.8%) received radical mastectomy, 27 participants (56.2%) received modified radical mastectomy, and another 12 participants (25%) received breast-conserving surgery.

There were no significant differences for average age, years of education, marital status, postoperative duration, duration of anxiety and depression, the baseline scores of PHQ-9, GAD-7, and PSQI among the three kinds of participants received different surgery.

### Comparison of Characteristics Between the Absentees Group and the iMBSR Group

As shown in Table 1, there were no significant differences for average age, marital status, years of education, postoperative duration, symptoms of anxiety and depression, operation methods among absentees group, partial attendees of iMBSR group, and completers of iMBSR group at the baseline.

**Table 1:** Comparison of characteristics among the control group and the part-time group (class number < 4) and full-time (class number ≥ 4) groups.

**Variable**	**Absentees group** **(*n* = 19)**	**Partial attendees** **(*n* = 9)**	**completers** **(*n* = 20)**	**Statistical value**	***P*-value**
Age (yr)	44.6 ± 4.2	45.1 ± 5.7	45.9 ± 5.4	*F* = −0.316	0.73
Education years	13.2 ± 1.5	14.4 ± 2.1	14.6 ± 2.1	*F* = −2.961	0.062
Marriage (married, %)	15 (78.9)	6 (66.7)	14 (70.0)	χ^2^ = 0.756	0.76
Postoperative duration (m)	9.0 ± 2.3	7.8 ± 3.3	8.5 ± 2.3	*F* = 0.781	0.46
Duration of anxiety and depression (m)	3 [2]	4 [3]	3 [1]	*H* = 1.770	0.41
Operation methods (%)				χ^2^ = 3.120	0.54
Radical	5 (26.3)	1 (11.1)	3 (15.0)		
Modified	10 (52.6)	7 (77.8)	10 (50.0)		
Conservative	4 (21.1)	1 (11.1)	7 (35.0)		
Class numbers	0	2.1 ± 0.8	7.8 ± 0.9	*H =* 46.14	<0.001^*^
Daily practice time (min)	0	13.3 ± 4.3	51.3 ± 21.6	*H =* 43.00	<0.001^*^
PHQ-9					
Baseline	15.7 ± 1.5	15 [2]	14 [3]	*H* = 5.989	0.050
Mid-intervention	16 [2]	12.8 ± 1.3	10.5 ± 3.0	*H* = 25.483	<0.001^*^
Post-intervention	15.1 ± 1.7	11.1 ± 2.6	6.6 ± 3.0	*H* = 34.987	<0.001^*^
GAD-7					
Baseline	15.5 ± 1.4	14.9 ± 1.9	13.3 ± 2.4	*F* = 6.225	0.004^*^
Mid-intervention	14.8 ± 1.5	12.2 ± 2.0	9.8 ± 2.9	*H* = 26.195	<0.001^*^
Post-intervention	14.4 ± 1.3	10.1 ± 3.8	6.7 ± 3.2	*H* = 31.787	<0.001^*^
PSQI					
Baseline	12.4 ± 1.5	12.6 ± 4.1	11.7 ± 2.9	*H* = 1.841	0.40
Mid-intervention	11.8 ± 1.8	9.8 ± 1.2	8.0 ± 2.7	*H* = 19.209	<0.001^*^
Post-intervention	11.4 ± 1.6	7.3 ± 2.1	5.2 ± 2.4	*F* = 45.750	<0.001^*^
Reduction rate					
PHQ9- Mid-intervention	4.1 ± 6.5%	18.0 ± 8.4%	25.5 ± 12.6%	*H* = 27.236	<0.001^*^
PHQ9- Post-intervention	4.1 ± 5.2%	28.5 ± 21.4%	53.3 ± 19.2%	*H* = 35.631	<0.001^*^
GAD7- Mid-intervention	4.0 ± 5.7%	17.7 ± 11.7%	27.6 ± 12.4%	*H* = 27.550	<0.001^*^
GAD7- Post-intervention	6.7 ± 3.4%	31.8 ± 25.8%	51.8 ± 18.2%	*H* = 32.786	<0.001^*^
PSQI- Mid-intervention	4.5 ± 5.2%	17.1 ± 19.1%	32.5 ± 14.5%	*H* = 29.242	<0.001^*^
PSQI- Post-intervention	7.8 ± 5.7%	32.3 ± 26.8%	56.3 ± 15.6%	*H* = 33.029	<0.001^*^

*Data are presented as mean ± SD, median [interquartile range] or absolute numbers (percentage)*.

*Reduction rate = (baseline score – score after intervention)/baseline score × 100%*.

* *indicate the P value has the statistical significance*.

From baseline to post-intervention, there were significant differences for scores and reduction rates of PHQ-9, GAD-7, and PSQI within iMBSR group, but these significant differences didn't be found in absentees group.

At baseline, there was significant difference in the scores of PHQ-9 and GAD-7 between the absentees group and iMBSR group; at mid-intervention and post-intervention, the differences from scores of PHQ-9, GAD-9, and PSQI between absentees group and iMBSR group became more significant than baseline. The scores of PHQ-9, GAD-7, and PSQI in the absentees group were significantly higher than those of the iMBSR group. These changes were not only demonstrated by the original scores, but also reflected on the reduction rate. The reduction rates of PHQ-9, GAD-7, and PSQI in iMBSR group at mid-intervention and post-intervention were significantly higher than those in the absentees group.

### Comparison of Characteristics Between Partial Attendees of iMBSR Group and Completers of iMBSR Group

Table 2 showed that there were significant differences regarding the role of daily practice time in scores of PHQ-9, GAD-7, and PSQI between the partial attendees and completers of iMBSR group. In partial attendees of iMBSR group, the daily practice time was significantly shorter; scores of PHQ-9, GAD-7, and PSQI at mid-intervention and post-intervention were significantly higher than completers of iMBSR group. These changes were demonstrated in reduction rate as well. Reduction rates of PHQ-9 and GAD-7 at post-intervention, reduction rates of PSQI at mid-intervention and post-intervention in completers of iMBSR group were significantly higher than those in partial attendees of iMBSR group. Within the iMBSR group, scores of PHQ-9 (Cohen's *d* = 1.95, 95% CI: 1.30–2.56), GAD-7 (Cohen's *d* = 1.83, 95% CI: 1.30–2.36), and PSQI (Cohen's *d* = 1.87, 95% CI: 1.32–2.41) at post-intervention were lower than baseline. This represents large reductions of symptoms according to the rule of thumb of interpreting effect sizes (Cohen's *d* > 0.8 represents large effect size).

**Table 2:** Comparison of characteristics between partial attendees (class number < 4) and completers (class number ≥ 4) groups in iMBSR group (*n* = 29).

**Variable**	**Part-time** **(*n* = 9)**	**Full-time** **(*n* = 20)**	**Statistical value**	***P*-value**
Age (yr)	45.1 ± 5.7	45.9 ± 5.4	*T* = −0.335	0.74
Education years	14.4 ± 2.1	14.6 ± 2.1	*T* = −0.124	0.90
Marriage (married, %)	6 (66.7)	14 (70.0)	χ^2^ = 0.032	1.000
Postoperative duration (m)	7.8 ± 3.3	8.5 ± 2.3	*T* = −0.716	0.48
Duration of anxiety and depression (m)	4 [3]	3 [1]	*H* = 1.770-	0.41
Operation methods (%)			χ^2^ = 2.033	0.40
Radical	1 (11.1)	3 (15.0)		
Modified	7 (77.8)	10 (50.0)		
Conservative	1 (11.1)	7 (35.0)		
Daily practice time (min)	13.3 ± 4.3	51.3 ± 21.6	*T* = −0.530	<0.001^*^
PHQ-9				
Baseline	15 [2]	14 [3]	*H* = 2.711	0.100
Mid-intervention	12.8 ± 1.3	10.5 ± 3.0	*T* = 2.828	0.009^*^
Post-intervention	11.1 ± 2.6	6.6 ± 3.0	*T* = 3.853	0.001^*^
GAD-7				
Baseline	14.9 ± 1.9	13.3 ± 2.4	*T* = 1.744	0.093
Mid-intervention	12.2 ± 2.0	9.8 ± 2.9	*T* = 2.247	0.033^*^
Post-intervention	10.1 ± 3.8	6.7 ± 3.2	*T* = 2.547	0.017^*^
PSQI				
Baseline	12.6 ± 4.1	11.7 ± 2.9	*T* = 0.687	0.50
Mid-intervention	9.8 ± 1.2	8.0 ± 2.7	*T* = 2.545	0.017^*^
Post-intervention	7.3 ± 2.1	5.2 ± 2.4	*T* = 2.367	0.025^*^
Reduction rate				
PHQ9- Mid-intervention	18.0 ± 8.4%	25.5 ± 12.6%	*T* = −1.635	0.114
PHQ9- Post-intervention	28.5 ± 21.4%	53.3 ± 19.2%	*H* = −3.294	0.005^*^
GAD7- Mid-intervention	17.7 ± 11.7%	27.6 ± 12.4%	*T* = −2.018	0.054
GAD7- Post-intervention	31.8 ± 25.8%	51.8 ± 18.2%	*T* = −2.404	0.023^*^
PSQI- Mid-intervention	17.1 ± 19.1%	32.5 ± 14.5%	*T* = −2.391	0.024^*^
PSQI- Post-intervention	32.3 ± 26.8%	56.3 ± 15.6%	*H* = 3.930	0.047^*^

*Data are presented as mean ± SD, median [interquartile range] or absolute numbers (percentage)*.

*The PHQ-9 (Cohen's d = 1.95, 95% CI: 1.30–2.56), GAD-7 (Cohen's d = 1.83, 95% CI: 1.30–2.36) and PSQI (Cohen's d = 1.87, 95% CI: 1.32–2.41) scores of March were significantly lower than those of January in the iMBSR group*.

* *indicate the P value has the statistical significance*.

### Association of Therapeutic Effects on Reduction Rates of PHQ-9, GAD-7, PSQI With Daily Practice Time Within iMBSR Attenders

The differences among the subgroups in iMBSR were further analyzed, shown in Table 3. Similarly, scores of PHQ-9 and PSQI, reduction rates of PHQ-9 at post-intervention, scores of GAD-7, reduction rates of GAD-7 and PSQI at mid-intervention and post-intervention, had significant differences among the three subgroups.

**Table 3:** Clinical characters and symptom changes according to daily practice time in the iMBSR group (*n* = 29).

	**<30 min** **(*n* = 10)**	**30–60 min** **(*n* = 11)**	**>60 min** **(*n* = 8)**	**Value of statistical test**	***p*-value**
Age	44.8 ± 5.4	47.1 ± 6.7	44.6 ± 2.9	*H* = 1.405	0.50
Education years	14.5 ± 2.0	14.6 ± 2.2	14.5 ± 2.3	*H* = 0.006	1.00
Marriage (married, %)	7 (70.0)	10 (90.9)	3 (37.5)	χ^2^ = 5.813	0.046^*^
Postoperative duration (m)	7.7 ± 2.9	9.0 ± 2.0	8.0 ± 2.7	*F* = 0.768	0.47
Duration of anxiety and depression (m)	3.6 ± 1.9	4.1 ± 1.9	3.3 ± 1.2	*H* = 0.727	0.70
Operation methods (%)				χ^2^ = 5.917	0.17
Radical	1 (10.0)	1 (9.1)	2 (25.0)		
Modified	8 (80.0)	7 (63.6)	2 (25.0)		
Conservative	1 (10.0)	3 (27.3)	4 (50.0)		
PHQ9					
Baseline	15.5 ± 1.8	13.0 ± 2.9	15.1 ± 1.9	*H* = 4.861	0.088
Mid-intervention	12.9 ± 1.2	10.0 ± 3.4	10.9 ± 2.6	*H* = 4.728	0.094
Post-intervention	11.1 ± 2.5	6.8 ± 3.3	5.8 ± 2.3	*H* = 11.763	0.003^*^
GAD7					
Baseline	15.0 ± 1.8	12.6 ± 2.3	13.9 ± 2.5	*H* = 5.449	0.066
Mid-intervention	12.5 ± 2.1	9.5 ± 3.0	7.6 ± 2.9	*F* = 4.361	0.023^*^
Post-intervention	10.4 ± 3.7	6.6 ± 3.2	6.0 ± 2.6	*H* = 7.492	0.024^*^
PSQI					
Baseline	12.6 ± 3.8	10.5 ± 2.5	13.1 ± 2.9	*F* = 2.024	0.15
Mid-intervention	9.9 ± 1.2	7.2 ± 2.7	8.6 ± 2.5	*H* = 5.447	0.066
Post-intervention	7.7 ± 2.3	4.6 ± 2.2	5.3 ± 1.8	*F* = 6.237	0.006^*^
Reduction rate					
PHQ9-Mid-intervention	16.9 ± 8.6%	24.7 ± 14.2%	29.0 ± 8.9%	*F* = 2.753	0.082
PHQ9- Post-intervention	27.8 ± 16.9%	49.3 ± 20.2%	62.7 ± 12.0%	*H* = 11.914	0.003^*^
GAD7- Mid-intervention	16.5 ± 11.6%	27.1 ± 13.5%	30.9 ± 9.0%	*F* = 3.736	0.037^*^
GAD7- Post-intervention	30.4 ± 24.7%	50.7 ± 19.2%	57.4 ± 13.5%	*F* = 4.589	0.020^*^
PSQI- Mid-intervention	17.0 ± 18.0%	32.5 ± 16.0%	34.6 ± 12.5%	*F* = 3.530	0.044^*^
PSQI- Post-intervention	33.3 ± 26.0%	57.4 ± 14.6%	59.8 ± 10.2%	*F* = 6.079	0.007^*^

* *indicate the P value has the statistical significance*.

In Table 4, GLMs analysis showed that daily practice time was positively related to reduction rates of PHQ-9, GAD-7, and PSQI at post-intervention within the iMBSR group. In Table 5, for subgroup 1, the estimated marginal means of therapeutic effects (reduction rates of PHQ-9, GAD-7, and PSQI) were 26.6, 29.42, and 31.94%. For subgroup 2, the estimated marginal means of therapeutic effects were 50.87, 52.17, and 59.4% for depression, anxiety, and sleep quality, respectively. For subgroup 3, the estimated marginal means of therapeutic effects were 62.07, 56.56, and 58.75% for depression, anxiety, and sleep quality, respectively. Subgroup 1 had the lowest therapeutic effect among the three subgroups; meanwhile there were no significant differences in the reduction rates of PHQ-9, GAD-7, and PSQI at post-intervention between subgroup 2 and subgroup 3.

**Table 4:** Association of therapeutic effects on reduction rates of PHQ-9, GAD-7, and PSQI with daily practice time in iMBSR group by GLMs.

**Parameters**	**Antidepressant effect**		**Antianxiety effect**		**Anti-insomnia effect**	
	**B**	***P***	**B**	***P***	**B**	***P***
Daily practice time (min)	0.005	<0.001^*^	0.004	0.010^*^	0.004	0.010^*^
Postoperative duration (m)	−0.013	0.312	−0.009	0.584	−0.012	0.41
Duration of anxiety and depression (m)	−0.002	0.919	−0.008	0.735	−0.008	0.71

*Antidepressant effect, antianxiety effect and anti-insomnia effect were evaluated by the reduction rates of PHQ-9, GAD-7, and PSQI scores, respectively. Daily practice time, postoperative duration and duration of anxiety and depression were the covariates in the GLMs*.

* *indicate the P value has the statistical significance*.

**Table 5:** Estimated marginal means of therapeutic effects (reduction rates) in the practice groups according to the GLMs.

**Practice groups**	**Antidepressant effect**	**Antianxiety effect**	**Anti-insomnia effect**
<30 min	26.60%	29.42%	31.94%
30–60 min	50.87%	52.17%	59.40%
>60 min	62.07%	56.56%	58.75%

*The covariates were set in the three models. Postoperative duration was set at 8.28 months and duration of anxiety and depression was set at 3.69 months*.

## Discussion

In this study, 48 survivors of BC with symptoms of depression, anxiety, and sleep disturbance were recruited to the study. From baseline to post-intervention, the scores of depression, anxiety and sleep disturbance decreased in all survivors, especially for those participants who attended the iMBSR. What is more, the therapeutic effect of iMBSR for survivors of BC was positively correlated with the level of engagement of practice in terms of average daily practice time.

For symptom severity of depression, anxiety and sleep quality, there were no significant differences among different kinds of cancer treatment for survivors of BC at baseline. A large proportion of cancer survivors experience poor quality of life, anxiety, distress, fear of recurrence and lower levels of social support, psychological and social needs, and difficulty in coping (33). Therefore, these results suggest that no matter the type of treatment, it is important for early assessment of the psychological status for survivors of BC. In addition to the impact of cancer and surgical factors on emotions, the COVID-19 epidemic might also have contributed to depression, anxiety, sleep problems among these patients during the pandemic, during which strong emotional reactions were a common public phenomenon globally (16).

In our study, from baseline to post-intervention, for the decreased scores of depression, anxiety and sleep disturbance, there was no significant difference for 19 subjects who neither attended the iMBSR course nor had a daily practice. Within the iMBSR group, improvement was accomplished in the status of depression, anxiety and sleep quality since mid-intervention among all 29 participants. Not only the scores of PHQ-9, GAD-7, PSQI decreased, but also the reduction rates of PHQ-9, GAD-7, PSQI increased significantly, indicating that internet-based MBSR could improve emotional well-being and sleep qualify among survivors of BC. A large randomized trial showed that adapted 6-week MBSR had short-term effectiveness for the psychological symptoms, could reduce salivary cortisol and pro-inflammatory cytokine interleukin-6 (IL-6) levels during the 6 weeks (34), and could modulate tumor necrosis factor α (TNFa) and IL-6 during 6 to 12 weeks rather than during the MBSR training period in survivors of BC (35). A randomized controlled trial using 8-week MBSR showed that MBSR had potential for alleviating depression, symptom experience, and for enhancing coping capacity, mindfulness and posttraumatic growth, and led to beneficial effect on immune function (36). Another 8-week MBSR for survivors of BC reported persistent benefits with reduced anxiety, depression, and improved mental health quality of life over 24 months of follow-up (37). Our results supported the notion that likes in-person MBSR, iMBSR is an effective intervention for reducing adverse psychological symptoms associated with cancer diagnosis or treatment among survivors of BC.

In this study, we also found that both the scores and reduction rates of PHQ-9, GAD-7, and PSQI had significant differences between the partial attendees and completers of iMBSR program, indicating that the completion of 8-week iMBSR course would be better and more suitable for the relief of depression, anxiety and sleep quality for survivors of BC. The reasons behind those difference might be due to participation and engagement which play an important role in outcomes for mindfulness based therapies (38), however, the specific mechanism between the partial attendees and completers of MBSR is currently unclear. Among the differences, the reduction rate of PSQI at mid-intervention in completers of iMBSR group was already significantly higher than those in partial attendees of iMBSR group, and for completers of iMBSR group, their PSQI scores on average at post-intervention were 5.2, suggesting that sleep quality would benefit from iMBSR the most among all symptoms, and had faster effect than other symptoms. Therefore, poor sleep quality may serve as a particular motivator for mindfulness practice, leading to better outcomes Furthermore, our study raised and answered partially another question that whether different engagement level of iMBSR could produce different size of effectiveness, both psychologically and physically.

In the present study the scores of PHQ-9 and PSQI, reduction rates of PHQ-9 at post-intervention, scores of GAD-7, reduction rates of GAD-7 and PSQI at mid-intervention and post-intervention, had significantly differences among subgroups of practice time, indicating that survivors of BC who practiced more than 30 min daily had the better relief in depression, anxiety and sleep disturbance. GLMs analysis showed further that daily practice time was positively correlated with reduction rates of PHQ-9, GAD-7, and PSQI at post-intervention within iMBSR participants. Survivors of BC who practiced <30 min daily had the lowest therapeutic effects, and those practiced more than 60 min had the highest therapeutic effects. However, there were no significant differences for the reduction rates of symptoms at post-intervention between survivors of BC who practiced 30–60 min and those who practiced more than 60 min. Therefore, the results indicated that iMBSR training has a potential dose–response relationship, with a threshold of >30 min daily practice for most beneficial symptoms reduction. This interesting finding is not only consistent with the recommendation in a systematic review that courses should last at least 4 weeks; 30 min of practice for 6 days a week should be encouraged (38), but also clearly indicated that iMBSR training has a potential dose–response relationship.

There are a number of limitations of this current study. First, generalizability of the study findings is limited by the sample size. Recruitment period lasted for 3 months from November 1th 2019 to January 31th 2020, and there was a total of 48 BC patients recruited. However, recruitment was disrupted with the COVID-19 outbreak. Along with other concerns, only a total of 29 participated in the iMBSR. Another limitation is inadequate assessment for the subjects, such as the evaluation for the life quality, self-compassion and mindfulness which are regarded as important factors in mindfulness practice (39). During the epidemic, the assessments and training were administered and delivered only remotely *via* internet. Too many assessments *via* internet runs the risk of low quality of returned questionnaires, therefore only depression, anxiety, and sleep problems were assessed so as to ensure the quality of these outcome measures. Furthermore, no formal psychiatric diagnoses were made due to the lack of in-person interview during the pandemic. In addition, as noted in the results, nearly 40% of patients did not attend iMBSR courses or practice, which might have the self-selection bias in this sample. The mindfulness interventions had a wide range in dropout rates (7.7–52.3%) (38). Therefore, how to improve the participation rate and level of engagement is worth exploring, such as reducing the session number of MBSR and the daily practice time requirement, and utilizing the internet. The dropout rate presented in this study is a common issue that needs further improvement in internet-based mindfulness trials and internet-based psychological interventions in general (40). The major question was that patients who were absent were chosen as the control group. This group might be lack of motivation. However, we not only compared the iMBSR group with the control group, we also compared themselves from baseline to 2 month later. From baseline to post-intervention, there were significant differences for scores and reduction rates of PHQ-9, GAD-7, and PSQI within iMBSR group, but these significant differences didn't be found in absentees group. Of course, the absentee group was not a “true control” comparison but there was self-selection bias involved. That is, those who might be more likely to benefit from MBSR stayed and indeed showed improvement, and those who might be less likely to benefit from the intervention to start with due to various factors dropped out. So in future clinical trial, using a true randomized controlled trial is needed to further examine the efficacy of iMBSR among survivors of BC due to the design limitation of this study. The last question was that the impact of gender of the group instructor on effectiveness of the intervention is something warrants further research.

In summary, the current study found that survivors of BC have symptoms of depression, anxiety and sleep disturbance, especially during the period of COVID-19 outbreak, and iMBSR is an effective intervention for reducing these adverse psychological symptoms. Secondly, for survivors of BC, iMBSR practice has a potential dose–response efficacy, with a threshold of >30 min daily practice for most beneficial symptoms reduction.

## Data Availability Statement

The original contributions presented in the study are included in the article/supplementary material, further inquiries can be directed to the corresponding author/s.

## Ethics Statement

The studies involving human participants were reviewed and approved by the Ethics Committee of the Second Affiliated Hospital to Kunming Medical University. The patients/participants provided their written informed consent to participate in this study. Written informed consent was obtained from the individual(s) for the publication of any potentially identifiable images or data included in this article.

## Author Contributions

CK and JY performed data collection, data analysis, data interpretation, and manuscript preparation. ZY, JY, LX, and YW collected the data. XF conducted statistical analyses. SS carried out data interpretation, manuscript preparation, and language revision. HT made language revision. All authors contributed to the article and approved the submitted version.

## Funding

This work was supported by Grant 81660235 and 81971288 from the National Natural Scientific Foundation of China, and Grant 20TJBXKY06 from Special fund for fighting the COVID-19 outbreak sponsored by Tongji University School of Art and Media and Institute of Disaster Medicine, East Hospital, Tongji University. Work by Shufang Sun was supported by National Institute of Health (K23AT011173; PI: Sun).

## Conflict of Interest

The authors declare that the research was conducted in the absence of any commercial or financial relationships that could be construed as a potential conflict of interest.

## Publisher's Note

All claims expressed in this article are solely those of the authors and do not necessarily represent those of their affiliated organizations, or those of the publisher, the editors and the reviewers. Any product that may be evaluated in this article, or claim that may be made by its manufacturer, is not guaranteed or endorsed by the publisher.
